# Chemistry as biology by design

**DOI:** 10.1111/1751-7915.13345

**Published:** 2018-11-28

**Authors:** Kristala L. J. Prather

**Affiliations:** ^1^ Department of Chemical Engineering Microbiology Graduate Program Synthetic Biology Center at MIT Massachusetts Institute of Technology Cambridge MA 02139 USA

On 3 October 2018, Prof. Frances H. Arnold of the California Institute of Technology (Pasadena, USA) was announced as a recipient of the Nobel Prize in Chemistry for her pioneering work on directed evolution. Her corecipients, George P. Smith of the University of Missouri in Columbia (USA) and Sir Gregory P. Winter of the MRC Laboratory of Molecular Biology in Cambridge (United Kingdom), developed phage display and used this approach to evolve antibodies with enhanced binding properties, respectively. The recognition of Arnold with Smith and Winter may lead one to believe that her work has focused on medical applications. However, the Nobel committee lists as the prize motivation: ‘for the directed evolution of enzymes’.^1^ Enzymes, nature's catalysts, are essential for molecular transformations, and the ability to radically alter – or even create – their catalytic function is key to the true emergence of a bioeconomy, built upon principles of renewability and sustainability.

It is no secret that Biology is quite a remarkable Chemist. The rich history of natural products as therapeutic agents provides ample evidence of the exquisite ability of enzymes to break and make chemical bonds, ultimately forming complex structures such as those found in antibiotics and antifungals that have proven essential for human health and development. While many of these molecules are accessible through synthetic organic chemistry, most are not practically so. That is, the large number of reaction steps and low overall yields of chemical synthesis make fermentation the preferred method of production. Beyond natural products, other biochemicals have been efficiently produced commercially through fermentation, including various amino acids (Wendisch *et al*., 2016), citric and lactic acids (Chen and Nielsen, 2016) and, of course, the old standby ethanol. In some cases, like ethanol, little manipulation of the microbial host is required to achieve acceptable production metrics, with process design as the only engineering input to the system. In others, metabolic engineering comes into play, through which organisms (typically microbes) are modified to increase product titre (i.e. concentration), rate (i.e. productivity, in units of concentration per time) and yield (i.e. fraction of available substrate converted to the target product). All of these molecules, however, have one thing in common: they are all naturally formed through established biosynthetic pathways.

Does this mean that only those compounds first identified as natural biological metabolites are amenable to the fermentation‐based approach to chemical synthesis? Of course not – that would be too boring! Enzymologists have known for years that biocatalysts do not conform to the one‐lock/one‐key model often presented in first‐year biochemistry courses. This knowledge has been exploited to produce a number of molecules through single‐step reactions, perhaps most notably in the synthesis of pharmaceuticals (Bornscheuer *et al*., 2012). The plasticity of enzymes has also been exploited in complete metabolic pathways, enabling the design of novel biosynthetic routes (Atsumi *et al*., 2008; Martin *et al*., 2013). In many cases, however, the catalytic activity of the enzyme when working on a non‐native substrate is too low to be practically useful. Enter directed enzyme evolution (Zhang *et al*., 2010). Computational protein design has even made it possible to create enzymes that perform reactions *not* found in nature (Rothlisberger *et al*., 2008). Perhaps, not surprisingly, these enzymes frequently perform quite poorly. What to do to make them stronger, faster, better? Directed evolution (Khersonsky *et al*., 2010)! Proceeding directly to evolution has also enabled the construction of an enzyme that performs a reaction not found in nature, one that forms bonds between carbon and silicon, two earth‐abundant elements (Kan *et al*., 2016).

Taken together, what do these technological advancements already achieved and those on the horizon foretell? The industrial chemist of the future may well view the microbial cell as the default reaction vessel. She can decide what molecule she wants to produce – one that may be a promising new therapy against a human disease or that may provide biodegradable materials with net neutral carbon footprint. She draws the structure in the graphical user interface of software that will turn and churn and spit out several possible synthetic routes, all starting from simple carbon sources like glucose, glycerol or xylose. (Of course, she used an electronic pen to draw the structure, which is immediately cleaned up and reproduced with perfectly positioned bond lengths and angles.) The routes generated by the algorithm present a step‐by‐step biosynthetic scheme augmented by recommendations for specific enzymes to perform each reaction. The algorithm compares known substrates for each enzyme to the proposed substrate and rank orders the recommendations. Whole pathways are subsequently ranked by taking into consideration the feasibility of each individual step, as well as the thermodynamics of the pathway. Where no known enzyme exists, the algorithm suggests a *de novo* scaffold design to create the enzymatic activity desired. When the chemist chooses her top pathway (or two or three), she pushes that selection through to the biofoundry (Chao *et al*., 2017), where genes are synthesized and assembled into expression constructs (lest we forget our promoters, ribosome binding sites and terminators) before being introduced into a handful of host organisms. The genetic constructs encoding each enzyme might be introduced and tested one at a time or, feeling lucky, she might decide to go for the home run right away. Unfortunately, she is unlikely to find that one design produces the target compound at the right titre, rate and yield, but she is neither disappointed nor dismayed. She knows that evolution is on her side. She creates a biosensor that can readily detect the target molecule (Zhang *et al*., 2015), whether an intermediate or end product, and starts the first of several rounds of directed evolution. The lessons learned from each round, combined with insights from the crystal structures of promising variants, feed into the iterative design process. Armed with additional tools from the Design‐Test‐Build‐Learn cycle of synthetic biology and not a small number of robotic platforms for construction and manipulation of microbial strains, it is only a matter of months before she reaches her objective. Finishing early enough to enjoy an afternoon of relaxation in celebration, she starts thinking ahead to the next project, wondering where is the true limit of Chemistry as Biology by design.

## Note

1 https://www.nobelprize.org/prizes/chemistry/2018/arnold/facts/
